# Preparation of manuscript

**DOI:** 10.4103/0971-6203.58774

**Published:** 2010

**Authors:** A. S. Pradhan

**Affiliations:** JMP, AMPI, C/o RP and AD, CT and CRS, Anushaktinagar, Mumbai - 400 094, India. E-mail: editor@jmp.org.in

It is a great pleasure to share the progress of the Journal of Medical Physics (JMP) of the Association of Medical Physicists of India (AMPI), during the last four years, since the launch of its online version in the first quarter of 2006.[1] It may be noted that more than 120 manuscripts were received during 2009 from scientists the world over as compared to 37 manuscripts received during 2006. On the punctuality, the issue of any quarter was published well before the end of the each quarter after the publication of the first issue of 2006. Of late the JMP issues are brought out in the beginning of the assigned quarter. The journal has improved in punctuality and quality[2] and taken a big leap forward by going on PubMed and PMC. This has enhanced the onus on the Editorial Board, in turn on authors, to ensure that JMP meets the requirements of an international journal.

This editorial on the preparation of manuscript is intended to help the young authors (specially the novice or amateur authors) so that their work is properly presented and the referees and the editors do not struggle to search for the relevance and the originality of the work. It is important that a good work should not appear bad due to inapt presentation and get into revisions leading to unwanted delay in acceptance and publication. The points discussed here are in addition to the “Instructions to authors” appearing in the end of this issue, which have to be adhered strictly. We hope that this Editorial will be read by the aimed authors who intend to publish their research work in JMP.

## Theme and Language

A scientific paper is a written and published report describing original research results. The theme should be properly worked out. To write a paper, it is not always necessary to have arrived at a new technique, theory, model, etc; sometimes, a negative result could also become as important as a positive result. One has to keep in mind that a manuscript is intended to be read by the scientific community where the precise choice of words is important such that no other unintended meaning could be derived. Lack of clarity in the statements is one of the more common reasons for manuscript rejection. It is advisable to avoid complex ways of saying a simple thing and to keep the sentences short. Literary language is not required. It is important to stick to the use of either past or present tense and remain consistent. Past tense is preferred to describe materials and methods or experimental and the results whereas conclusion could be in present tense. Subheadings are very useful and help in keeping the issues separate. Paragraphs are important to break the text up into readable units.

## Need of Advance Planning

A publication could be the outcome of a preplanned research project or planned after realizing that the byproduct of some work would lead to a good publication. In either case much of the thought process of composing a manuscript takes place during the period of the design of the work based on review of the concerned literature, well before the writing process actually begins. Proper focusing on the parameters of the work is critical in determining the likelihood that the resultant manuscript would make an acceptable publication as per scope of the journal. During the course of work, it has to be borne in mind that sufficient and statistically significant data have to be made available to support statements in the results and figures and tables.

## Writing of Text

There is no single way to be the best way to start the writing of a manuscript as it varies from manuscript to manuscript and also on type e.g. Review Article, Original Article, Scientific or Technical Note, Book Review, Letter to the Editor, Case Report etc. The basic needs of a publication remain the same in almost all the cases. A research paper normally follows the text in a sequence of title of paper, names of author/s, affiliation of author/s with address/ess, abstract, introduction including principles and theoretical approach, materials and methods or experimental, results, discussion, conclusions, references, acknowledgements, tables, figures etc. In general, the writing of a manuscript should start after finalization of the results. Thus a section on ‘Results’ or ‘Results and Discussion’ is the first to be completed. At this stage all the results need to be compiled and self-reviewed. The deficiencies have to be recognised and need of additional experiments and review of literature to defend the hypothesis is to be met. It may be borne in mind that all the data obtained during the study need not be forcibly included. Only the results essential to demonstrate and support an outcome be included in an effective way and the main results may form tables or figures.

Title must be short with fewest possible words, adequately indicative of the contents, keeping a big picture in mind. In the ‘Discussions’ section (if it is separate from ‘Results’), repetition of what is written under ‘Results’ should be avoided; no speculative statements should be included for which no supportive data is presented / demonstrated / cited. ‘Materials and Methods’ or ‘Experimental’ section may then be finalized by meticulously providing all the important details of the experiments / methods or formulism. Next could be the section of ‘Introduction’ which is the most important part of the manuscript. It must provide the rational or the need of the work, its importance related to the work already done in the field and statements on foreseen outcome of the work presented in the manuscript. Aim of the study must be given in plain and straight forward language.

A declaration on the expected outcome of the work in the ‘Introduction’ is critical for reviewers to understand and judge on what was aimed and what is achieved. Citation of reference at appropriate places is also very important. This not only gives credit to the others in the field but also adds to the creditability of the work of the authors and help readers to find further information. Care should be taken to avoid confusion as to what is achieved by the authors and what has been done by others. A doubt of plagiarism of any type (including copying of ones own published paper) leads to a negative decision and other complications.

Although the ‘Abstract’ is the first part of the manuscript, it is advisable to write it in the end after finishing all the other sections. Sections of ‘Conclusions’ and the ‘Abstract’ could be written simultaneously as some of the contents of these two sections are similar. ‘Abstract’ has to be very brief, 100-250 words, to summarize the purpose, methods, results and conclusions. It should be able to stand alone because abstract is a main bibliographical source. ‘Conclusions’ should concentrate on the technical outcome of the study. Tables should be self-explanatory with self sustainable captions. Figures (with captions) should be clear enough and the graphs should have labels on X and Y axes (with units, as and where possible). A section on ‘Acknowledgment’ is generally optional but it is better to mention the names of agencies providing the financial support and those who contributed to the work and are not coauthors.

## Review Process

The authors should not get discouraged if asked to revise manuscripts. Referees generally make all efforts to improve on the manuscript by the process of revision and re-revision and their comments must be taken very seriously. It is important for the authors to respond to the referees' comments and to submit the revised version along with a report (Author Comment File) indicating point to point reply to the comments of the referees. If the authors have a difference of opinion with the referee, the same could be included for a healthy scientific interaction between the authors and the peers / referees. Usually, full opportunity is provided to the authors to defend their contribution. The final decision on the manuscript is taken by the Editor after reviewing the comments of the referees, the response of the authors to the comments and the revised version.
